# Geographically driven differences in microbiomes of *Acropora cervicornis* originating from different regions of Florida’s Coral Reef

**DOI:** 10.7717/peerj.13574

**Published:** 2022-06-16

**Authors:** Sara D. Williams, J. Grace Klinges, Samara Zinman, Abigail S. Clark, Erich Bartels, Marina Villoch Diaz Maurino, Erinn M. Muller

**Affiliations:** 1 Mote Marine Laboratory, Sarasota, FL, United States of America; 2 Mote Marine Laboratory, Elizabeth Moore International Center for Coral Reef Research & Restoration, Summerland Key, FL, United States of America; 3 Nova Southeastern University, Dania Beach, FL, United States of America; 4 The College of the Florida Keys, Key West, FL, United States of America

**Keywords:** Microbiome, *Acropora cervicornis*, Staghorn coral, *Aquarickettsia*, Coral Restoration, Florida’s Coral Reef, *Spirochaeta*

## Abstract

Effective coral restoration must include comprehensive investigations of the targeted coral community that consider all aspects of the coral holobiont—the coral host, symbiotic algae, and microbiome. For example, the richness and composition of microorganisms associated with corals may be indicative of the corals’ health status and thus help guide restoration activities. Potential differences in microbiomes of restoration corals due to differences in host genetics, environmental condition, or geographic location, may then influence outplant success. The objective of the present study was to characterize and compare the microbiomes of apparently healthy *Acropora cervicornis* genotypes that were originally collected from environmentally distinct regions of Florida’s Coral Reef and sampled after residing within Mote Marine Laboratory’s *in situ* nursery near Looe Key, FL (USA) for multiple years. By using 16S rRNA high-throughput sequencing, we described the microbial communities of 74 *A. cervicornis* genotypes originating from the Lower Florida Keys (*n* = 40 genotypes), the Middle Florida Keys (*n* = 15 genotypes), and the Upper Florida Keys (*n* = 19 genotypes). Our findings demonstrated that the bacterial communities of *A. cervicornis* originating from the Lower Keys were significantly different from the bacterial communities of those originating from the Upper and Middle Keys even after these corals were held within the same common garden nursery for an average of 3.4 years. However, the bacterial communities of corals originating in the Upper Keys were not significantly different from those in the Middle Keys. The majority of the genotypes, regardless of collection region, were dominated by Alphaproteobacteria, namely an obligate intracellular parasite of the genus *Ca.* Aquarickettsia*.* Genotypes from the Upper and Middle Keys also had high relative abundances of *Spirochaeta* bacteria. Several genotypes originating from both the Lower and Upper Keys had lower abundances of *Aquarickettsia*, resulting in significantly higher species richness and diversity. Low abundance of *Aquarickettsia* has been previously identified as a signature of disease resistance. While the low-*Aquarickettsia* corals from both the Upper and Lower Keys had high abundances of an unclassified Proteobacteria, the genotypes in the Upper Keys were also dominated by *Spirochaeta*. The results of this study suggest that the abundance of *Aquarickettsia* and *Spirochaeta* may play an important role in distinguishing bacterial communities among *A. cervicornis* populations and compositional differences of these bacterial communities may be driven by regional processes that are influenced by both the environmental history and genetic relatedness of the host. Additionally, the high microbial diversity of low-*Aquarickettsia* genotypes may provide resilience to their hosts, and these genotypes may be a potential resource for restoration practices and management.

## Introduction

Caribbean populations of the staghorn coral *Acropora cervicornis* have exhibited marked declines since 1980 due to a multitude of stressors including infectious disease, poor water quality, high sea surface temperatures, and overfishing (*Acropora* Biological Review Team, 2005). Once a predominant reef-building species of the Western Atlantic and Caribbean, this coral species has experienced a population reduction of nearly 95% and is now listed as critically endangered under the International Union of Conservation of Nature’s (IUCN) Red List (Aronson & Precht, 2006; *Acropora* Biological Review Team, 2005). The decline of this species, in concurrence with its sister species, *A. palmata*, has contributed to a reduction in Caribbean coral reef cover of up to 80% (Gardner et al., 2003). Asexual propagation of *A. cervicornis*, for coral gardening purposes in both land and *in situ* nurseries, and restoration to *in situ* reef environments, referred to here as ‘outplanting’, is a commonly used conservation strategy on Florida’s Coral Reef (Schopmeyer et al., 2017; Lirman et al., 2010) and throughout the greater Caribbean (Mercado-Molina, Ruiz-Diaz & Sabat, 2015; Young, Schopmeyer & Lirman, 2012). Despite the overall success of outplanting (Boström-Einarsson et al., 2020; Schopmeyer et al., 2017), environmental conditions have not improved and the ability for outplants to survive long-term is questionable (Ware et al., 2020; van Woesik et al., 2021), therefore, restoration efforts must thoughtfully integrate genotypes most likely to succeed and survive.

The use of microsatellite loci to distinguish different genotypes (or asexual clones) of *Acropora cervicornis* has not only revealed the high level of genetic diversity of this species within the Caribbean, but has also identified variation in traits relevant to restoration success. *A. cervicornis* populations are extensively structured at regional scales on Florida’s Coral Reef (FCR), with high variation in genetic diversity among regions (Drury, Manzello & Lirman, 2017). Significant differences in growth rates, frequency of branching, and calcification across coral genotypes have been documented in *Acropora* species (Lirman et al., 2014; Kuffner et al., 2017; Lohr & Patterson, 2017). Specific genotypes have been identified that are more resilient to stressors, including thermal stress (Drury et al., 2017; Lohr & Patterson, 2017; Ladd et al., 2017) and disease (Libro & Vollmer, 2016; Muller, Bartels & Baums, 2018). The complex association of the coral microbiome with host health state, however, precludes definitive association of preferential phenotypes with host traits alone. Symbiont identity plays an important role in determining thermotolerance (Swain et al., 2016; Cunning et al., 2015), disease response (Rouzé et al., 2016), and growth rates (Cunning et al., 2015) in *Acropora* species. Similarly, the bacterial communities harbored by corals perform numerous services for their hosts such as nutrient cycling and provide essential settlement cues (Lesser et al., 2007; Sharp, Distel & Paul, 2012; Peixoto et al., 2017). Certain coral-associated bacterial genera provide an essential first line of defense against pathogenic species, and therefore disease, through the production of antimicrobial compounds (Krediet et al., 2013; Glasl, Herndl & Frade, 2016). The diversity of the coral microbiome may also play a role in resilience to stressors, as higher microbial diversity may provide the coral with a greater arsenal of services to supplement coral metabolism and antibiotic defenses (Zilber-Rosenberg & Rosenberg, 2008; West et al., 2019).

Coral-associated microbes tend to vary by host species (Morrow et al., 2012; Littman et al., 2009; Rohwer et al., 2002), and within species, microbial community structure can be stable across broad geographic regions (Sunagawa, Woodley & Medina, 2010; Brener-Raffalli et al., 2018). Nonetheless, coral microbial communities respond to naturally-occurring spatiotemporal factors including depth (Glasl et al., 2017), predation by herbivores (Rice et al., 2019; Ezzat et al., 2020), and seasonality (Li et al., 2014; Ceh, Van Keulen & Bourne, 2011; Hong et al., 2009; Koren & Rosenberg, 2006). Further, the coral microbiome is responsive to environmental stressors including nutrient enrichment (Zaneveld et al., 2016; Wang et al., 2018), overfishing (Zaneveld et al., 2016; McDevitt-Irwin et al., 2017), macroalgal competition (Nugues et al., 2004; Vega Thurber et al., 2012; Pratte et al., 2018), and thermal stress (Bourne et al., 2008; Maher et al., 2019).

Florida’s Coral Reef has distinct spatial regions because of varying seawater circulation patterns due to the underlying geology that contribute to regional differences in environmental parameters and hardbottom communities (Jaap, 1984; Klein III & Orlando Jr, 1994; Murdoch & Aronson, 1999). While the reefs of the Upper Florida Keys (‘Upper Keys’) are fairly protected from the highly variable waters of the Gulf of Mexico and Florida Bay, reefs in the Middle and Lower Florida Keys (‘Middle Keys’ and ‘Lower Keys,’ respectively) are subjected to more variable conditions due to passes and tidal channels that allow for increased water exchange (Klein III & Orlando Jr, 1994). The regional delineations of Upper, Middle, and Lower Keys are used by conservation managers and practitioners to account for spatial differences in monitoring and restoration activities. Given the differences in environmental conditions and coral community structure among the regions of FCR, microbiomes of corals used in restoration throughout the Keys may differ based on nursery, and later, outplanting location, resulting in possible differences in restoration success.

We previously characterized the microbiomes associated with different genotypes of *A. cervicornis* identified as susceptible or resistant to diseases (Klinges et al., 2020). We found that microbiomes of disease-susceptible genotypes collected from the Lower Keys were characterized by an overwhelming dominance of the bacterial species *Candidatus* Aquarickettsia rohweri (Klinges et al., 2020; Klinges et al., 2022). In contrast, disease-resistant genotypes were characterized by a more even and diverse microbiome, with low abundances of *Ca.* Aquarickettsia (hereafter, *“Aquarickettsia*”; Klinges et al., 2020). Members of the genus *Aquarickettsia* are dominant in communities across many genotypes of *A. cervicornis* (Rosales et al., 2019; Gignoux-Wolfsohn et al., 2020) and a high abundance of its members are associated with increased disease prevalence, reduced coral growth, and increased tissue loss (Zaneveld et al., 2016; Shaver et al., 2017). *Ca.* Aquarickettsia rohweri was previously demonstrated to possess the genomic capacity to parasitize the coral holobiont for amino acids and ATP (Klinges et al., 2019) and responds positively to nutrient enrichment (Shaver et al., 2017; Klinges et al., 2022). Due to the apparent association of *Aquarickettsia* with disease-susceptible phenotypes, a broader assessment of the distribution of this bacterial genus across Floridian *Acropora* is necessary to examine its ubiquity and assess the contribution of *Aquarickettsia* to signatures of disease susceptibility and resistance. Comparison of acroporid microbiome composition across distinct geographic regions may allow for the identification of genotypes that are ideal candidates for restoration purposes, harboring low abundance of *Aquarickettsia* and therefore likely to be disease-resistant. Alternately, assessment of microbiomes from genotypes sourced from different regions of the Florida Keys may reveal that the relationship between *Aquarickettsia* and disease-response phenotype is limited to the Lower Keys.

Here, we examined differences in the microbial community composition of *Acropora cervicornis* genotypes originally collected from different regions of Florida’s Coral Reef and sampled after residing within Mote Marine Laboratory’s *in situ* coral nursery near Looe Key, FL (USA) for multiple years. Microbiomes of genotypes collected from the Upper, Middle, and Lower Keys were described using 16S rRNA high-throughput sequencing to characterize and compare the microbiomes of apparently healthy *A. cervicornis* residing within the coral nursery. We found that while the majority of *A. cervicornis* genotypes from all collection regions harbored microbiomes dominated by putative parasites of the genus *Aquarickettsia*, a subset of genotypes possessed a significantly different microbiome characterized by the dominance of either an unclassified sequence variant or the genus *Spirochaeta.* Additionally, genotypes from the Upper and Lower Keys retained a microbiome signature that was specific to the geographic region of origination, while genotypes from the Middle Keys were distinct from those originating from the Lower Keys, but not those from the Upper Keys. Our results provide key information on the diversity and potential geographical influences on the microbiomes of an important restoration species.

## Materials & Methods

### Sample collection and processing

**Figure 1:**
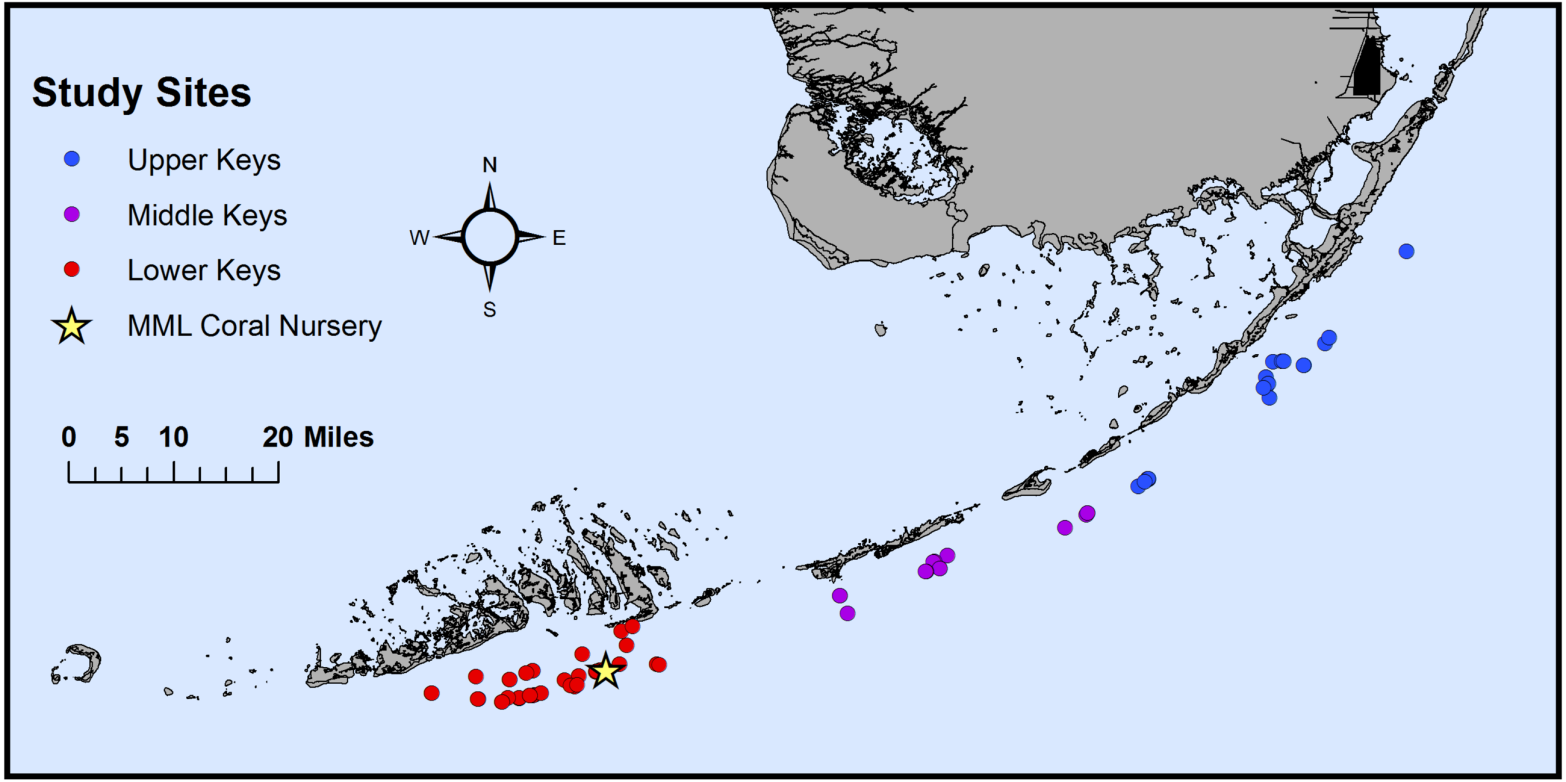
Map of the original collection locations of the sampled genotypes. Map of the original collection locations of the coral genotype fragments (*n* = 74) on Florida’s Coral Reef. Corals were originally collected from the Upper Keys (*n* = 19, blue), Middle Keys (*n* = 15, purple), and Lower Keys (*n* = 40, red) before being transferred to Mote Marine Laboratory’s (MML) *in situ* coral nursery (yellow star) near Looe Key, FL (USA).

In November-December 2019, *Acropora cervicornis* genotypes (*n* = 74) were collected from Mote Marine Laboratory’s *in-situ* nursery near Looe Key, FL (USA) in the Lower Keys and sampled for microbiome analysis. The Florida Keys National Marine Sanctuary authorized the use of nursery-grown corals under permit FKNMS-2015-163. These corals were curated for Mote’s coral restoration efforts throughout the Florida Keys and previously genotyped using both microsatellites (Baums, Miller & Hellberg, 2005) and the SNP chip platform (Kitchen et al., 2020). Of the 74 genotypes collected, 40 originated from the Lower Keys, 15 from the Middle Keys, and 19 from the Upper Keys (Fig. 1, Table S1). Genotypes from the Lower Keys had been in the nursery for the longest time, spending 8.1 ± 3.2 years (mean ± S.E.) there before sampling (Table S1), whereas genotypes from the Middle and Upper Keys spent 3.4 ± 0.7 and 3.3 ± 0.8 years, respectively, in the nursery before sampling (Table S1; see Supplemental Methods & Results for additional temporal analyses). The *A*. *cervicornis* fragments, each ranging between 1.5 and three cm in length, were placed in individual 2-oz Whirl-Paks containing ambient seawater for transport (approximately 35 min) to Mote’s Elizabeth Moore International Center for Coral Reef Research and Restoration (Summerland Key, FL, USA). Once back at the lab, the seawater in each Whirl-Pak was discarded and the fragments were flash-frozen in a dewar containing liquid nitrogen for one minute. The frozen samples were then immediately stored at −80 °C until further processing.

DNA was isolated from the frozen coral fragments using DNeasy PowerSoil Kits (QIAGEN, Germantown, MD, USA) with modifications to the manufacturer’s protocol (Rosales et al., 2020). Using sterile tweezers, 4–5 polyps were removed from every coral and transferred to DNeasy PowerBead tubes. During polyp excision, the corals were kept on ice to prevent thawing. Following DNA extractions, a NanoDrop One™ Microvolume UV-Vis Spectrophotometer (Thermo Fisher Scientific, Waltham, MA, USA) was used to quantify DNA concentrations and purity.

The bacterial communities of each sample were determined using 16S rRNA Illumina sequencing on the MiSeq platform. DNA was sent to MR DNA (http://www.mrdnalab.com, Shallowater, TX, USA) for barcoding, amplification, and sequencing. Amplification of the 16S rRNA gene variable region (V4) was conducted using primers 515F (GTGCCAGCMGCCGCGGTAA; Original Earth Microbiome Project; Caporaso et al., 2011) and 806R (GGACTACVSGGGTATCTAAT; Archaea 806R; Takai & Horikoshi, 2000). Barcodes were on the forward primer. A polymerase chain reaction (PCR; 30 cycles) was performed using the HotStarTaq Plus Master Mix Kit (QIAGEN, Germantown, MD, USA) under the following conditions: 94 °C for 3 min, followed by 28 cycles of 94 °C for 30 s, 53 °C for 40 s and 72 °C for 1 min, and by a final elongation step at 72 °C for 5 min. PCR products were checked on a 2% agarose gel to determine the success of amplification and the relative intensity of bands. Samples were pooled together in equal proportions based on their molecular weight and DNA concentrations. Pooled samples were purified using calibrated Agencourt Ampure XP beads (Beckman Coulter, CA, USA). Next, the pooled DNA library was generated using the Illumina TruSeq DNA library preparation protocol. Paired-end sequencing with a sequencing read length of 300 base pairs was performed at MR DNA using a single flow cell on a MiSeq following the manufacturer’s guidelines.

### Sequence data processing and analysis

All data processing and analysis were performed in the program R (version 4.0.3, R Development Core Team, 2019). A total of 24,392,378 reads across 74 samples were processed using DADA2 (v1.16; Callahan et al., 2016) in R (see Table S2 for reads retained at each step). After quality plot inspection, forward and reverse reads were truncated to 210 base pairs at their 3′ end. Sequences were truncated at the first position where a quality score was ≤ 2. Reads with a total expected error of >2 or with the presence of Ns were discarded. This resulted in a total of 21,770,005 reads. An initial total of 4,434 amplicon sequence variants (ASVs) were inferred from unique reads and paired-end reads were subsequently merged. ASVs that did not match a target length of 250–255 (1,542 ASVs) were discarded. A total of 776 two-parent chimeras (bimeras) were removed and taxonomy was assigned at 100% sequence identity using the Silva reference database (v132) to preserve the high resolution of ASV data (Quast et al., 2013). An average of 81.45% of initial reads, corresponding to 2,216 ASVs, were retained through the quality filtering pipeline. The Silva taxonomic classification for the genus MD3-55 was changed to *Candidatus* Aquarickettsia rohweri (hereafter, “*Aquarickettsia*”) for congruence with current identifications in the literature (Klinges et al., 2019). The ASV table resulting from DADA2 processing was imported into *phyloseq* (v1.30.0) (McMurdie & Holmes, 2013). A total of 71 ASVs taxonomically identified as chloroplasts, mitochondria, or eukaryotic sequences were removed (corresponding to 17,363, 82, and 56 reads, respectively). Taxa with less than 10 reads in 10% of the samples were removed, equating to 755 ASVs (4,641 reads). Using alpha rarefaction curves in *phyloseq* (Fig. S1), samples were rarefied to a minimum sequence depth of 26,537 reads, which allowed for the inclusion of all samples while still maximizing sample diversity.

### Diversity and differential abundance analyses

Non-metric Multidimensional Scaling analyses (nMDS) using the Bray-Curtis dissimilarity distances were performed to determine similarities in bacterial communities among samples due to collection region. Upon conducting the nMDS analysis, we visually identified 10 outliers. We then used the ‘OutlierDetection’ function (Outlier Detection package, Tiwari & Kashikar, 2019) to determine significant outliers out of all samples based on the euclidean distance method. Relative abundance plots confirmed differences between the non-outlier and outlier samples. All further analyses were done separately for both the non-outliers and outlier sample groups. nMDS analyses were repeated separately for both groups. Differences in beta diversity among collection regions of both the non-outliers and outliers were also tested using the betadisper function (vegan package; Oksanen et al., 2020) to calculate the multivariate homogeneity of dispersion of the Bray-Curtis distances. Pairwise comparisons were made using Tukey post-hoc comparison tests.

Differences in bacterial communities among samples due to collection region were tested using a permutational multivariate analysis of variance, PERMANOVA, of the Bray-Curtis dissimilarity (vegan package; Oksanen et al., 2020) for the rarefied dataset. Multiple pairwise PERMANOVA tests were used to compare beta diversity between collection regions. *P*-values were adjusted using the Bonferroni correction.

Alpha diversity as a function of collection region was assessed for the rarefied dataset using species richness and the Shannon diversity metric. Normality conditions were tested using Shapiro–Wilks tests. We used Kruskal-Wallis rank sum tests to determine significant differences in alpha diversity metrics by region. Additional pairwise Kruskal-Wallis rank sum tests with Bonferroni-corrected *P*-values were used to determine significant differences between collection regions.

To determine differentially abundant microbial taxa as a function of collection region, we used the R package corncob (Martin, Witten & Willis, 2021). Corncob uses beta-binomial regression models and accounts for varying sequencing depth and within-sample correlations between taxa (Martin, Witten & Willis, 2021). Using the un-rarefied data, we built our models using region as the predictor variable for both the non-outlier and outlier datasets separately.

## Results

### Microbiomes of *Acropora cervicornis* genotypes are characterized by differences in the relative abundance of the genus *Aquarickettsia*

**Figure 2:**
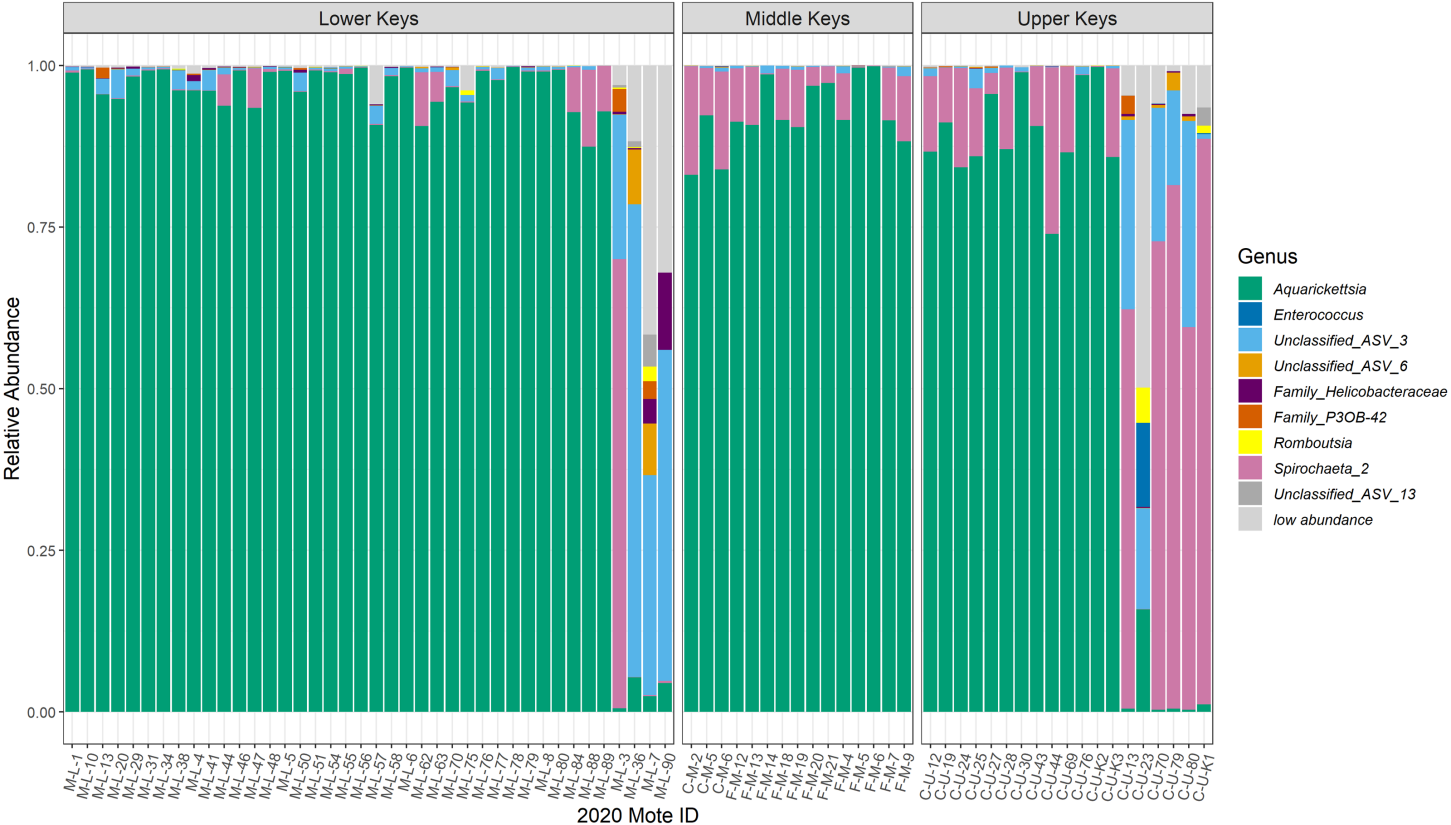
Relative abundance plot of all coral genotype fragments showing the genera of bacteria present with mean relative abundances over 0.001, separated by collection region. Relative abundance plot of all coral genotype fragments showing the genera of bacteria present with mean relative abundances over 0.001, separated by collection region. The ‘low abundance’ group represents all of the genera with relative abundance less than 0.001. The last four coral genotype fragments in the Lower Keys, and the last six coral genotype fragments in the Upper Keys are the outliers, or low-*Aquarickettsia* genotypes. There were no outliers in genotypes from the Middle Keys. According to NCBI’s basic local alignment tool (BLAST), ‘ASV 3’ is most similar to an uncultured bacterium clone plBB03 sampled from *A. palmata* in Puerto Rico (E =1e−127; 100% identity; GenBank EU861195.1), ‘ASV 6’ is most similar to an uncultured bacterium clone pl14H11 sampled from *A. palmata* in Puerto Rico (E =1e−127; 100% identity; GenBank EU853842.1), and ‘ASV 13’is most similar to an uncultured bacterium clone Apal_A03 from *A. palmata* (E =1e−127; 100% identity; GenBank GU118138.1).

Post-filtration, as described in the methods above, the dataset consisted of 1,195 ASVs, with a mean read depth per ASV of 1,643. The 16S rRNA sequences are available under BioProject ID PRJNA769275. Microbiomes of most genotypes were dominated by ASVs in the genus *Ca.* Aquarickettsia, except for genotypes identified as outliers due to the lower relative abundance of this taxon (Fig. 2). The average relative abundance of *Aquarickettsia* in non-outlier samples was 94.3 ± 5.51%, while in outlier samples it was 3.14 ± 4.83%. Of the *Aquarickettsia* ASVs, one strain in particular (ASV 1) dominated the microbiomes of most genotypes, accounting for approximately 90% or more of the relative abundances (Figs. S2 and S3). Many of the Lower Keys genotypes also had low relative abundances of bacteria in the genus *Spirochaeta*, which appeared in higher relative abundances in both the Middle and Upper Keys genotypes (Fig. 2). The average relative abundance of *Spirochaeta* in non-outlier samples was low and invariable, at 4.44 ± 5.72%, while in outlier samples it was highly variable at 43.2 ± 37.9% due to the low relative abundance of this genus in outlier samples from the Lower Keys, which were mostly dominated by an unclassified proteobacteria (‘Unclassified_ASV_3’). According to NCBI’s basic local alignment tool (BLAST), this ASV is most similar to an uncultured bacterium clone plBB03 sampled from *A. palmata* in Puerto Rico (E =1e−127; 100% identity; GenBank EU861195.1). The outlier genotypes in the Upper Keys were dominated by bacteria in the genera of both the unclassified proteobacteria and *Spirochaeta*. The relative abundance of this unclassified ASV was 0.78 ± 0.96% in non-outlier samples, and 29.3 ± 20.5% in outlier samples.

### Bacterial diversity significantly differed among genotypes initially collected from different regions and was higher in genotypes not dominated by *Aquarickettsia*

**Figure 3:**
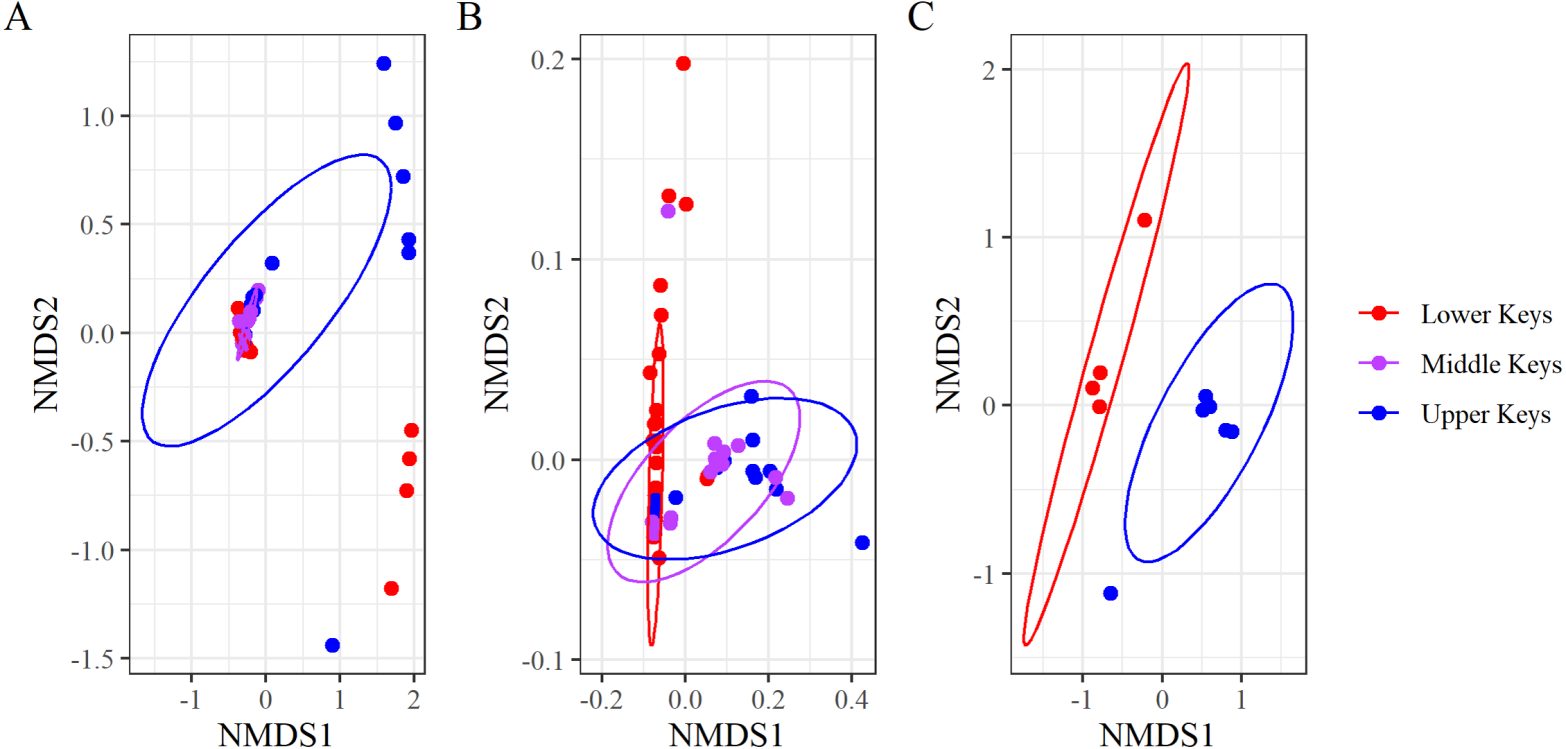
Non-metric multidimensional scaling analysis of the Bray–Curtis Distances grouping microbiome compositions by initial collection region. Non-metric multidimensional scaling analysis of the Bray–Curtis Distances grouping microbiome compositions by initial collection regions for (A) the full dataset (stress = 0.05), (B) the non-outlier samples (stress = 0.06), and (C) the outlier samples (stress = 0.09). Points represent samples, and the ellipses are 95% confidence ellipses for each collection region.

nMDS analysis for the full dataset (Fig. 3A) confirmed the existence of possible outliers. We identified 10 significant outliers of the 74 genotypes sampled: four from the Lower Keys and six from the Upper Keys. There were no outliers among genotypes sampled from the Middle Keys. These outliers matched the observations made through visualization of the relative abundances –bacterial communities of the non-outlier genotypes from all regions were dominated by *Aquarickettsia sp*., while the microbiomes of the outlier genotypes were composed of many highly relatively abundant taxa (Fig. 2). All further analyses were performed on both the non-outlier, or high-*Aquarickettsia*, and the outlier, or low-*Aquarickettsia*, datasets.

**Figure 4:**
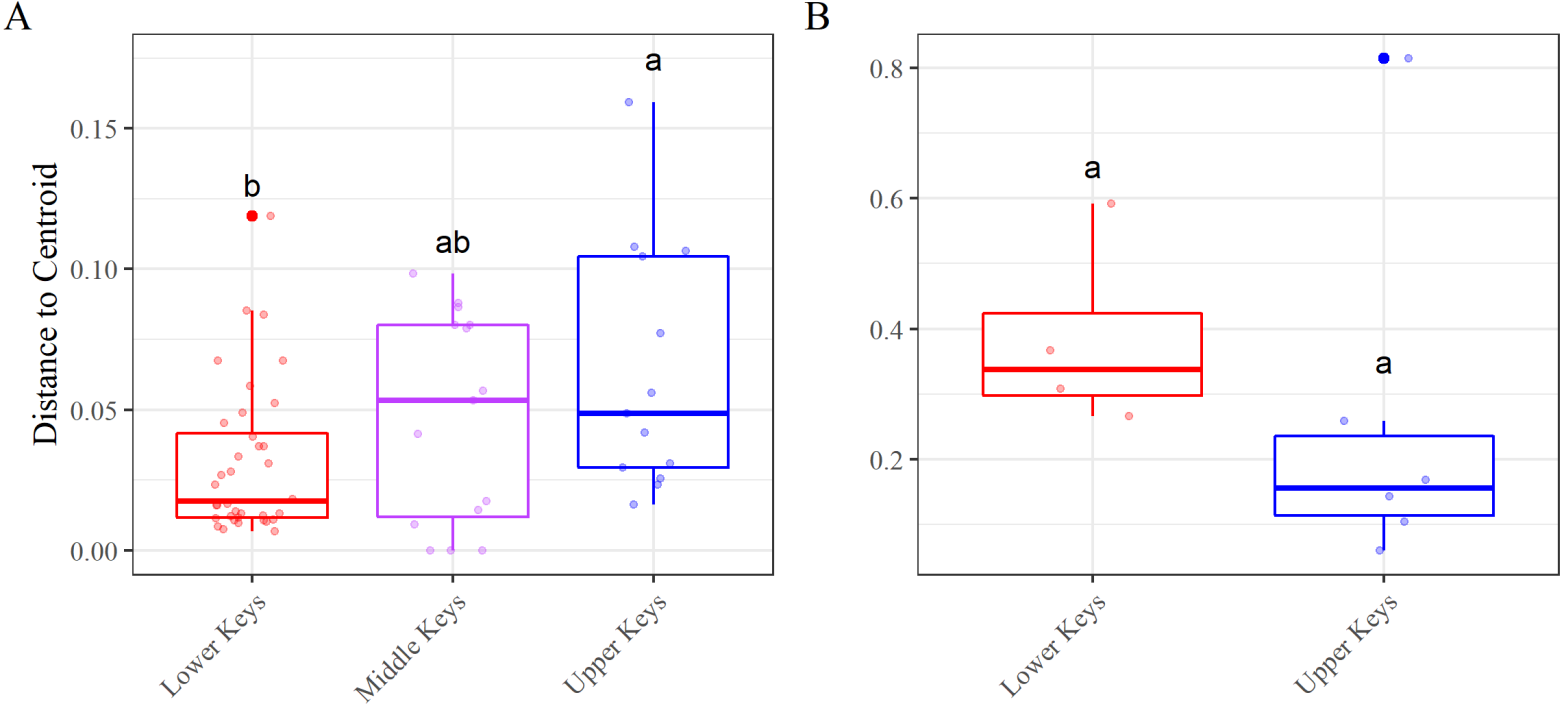
Beta diversity, using the betadisper function, results. Betadisper results for the (A) high-*Aquarickettsia* and (B) low-*Aquarickettsia* genotypes by collection region. Different letters denote significant differences according to the post hoc pairwise comparison tests.

Beta diversity analyses for both datasets determined significant differences among and between regions. nMDS analysis of the high-*Aquarickettsia* genotypes (non-outlier samples) demonstrated similarities among fragments by collection region (Fig. 3B), and was supported by the betadisper analysis that determined significant differences between Lower and Upper Keys genotypes, but not between the Middle Keys and the other two regions (Fig. 4A). Bacterial communities of the high-*Aquarickettsia* genotypes were significantly different by collection region (PERMANOVA, *df* = 2, *R*^2^ = 0.34, *F* = 0.05, *P* = 0.001; similar results for the full dataset in Table S3). Multiple PERMANOVA tests for the high*-Aquarickettsia* subset determined that the genotypes from the Lower Keys had significantly different microbial communities than both the Upper (*P* = 0.003) and Lower (*P* = 0.003) Keys; however, the microbial communities of the Upper and Middle Keys were not significantly different (*P* = 0.555; Table S3). The bacterial communities of the Lower and Upper Keys’ low*-Aquarickettsia* genotypes (outlier samples) were significantly different as shown by the 95% confidence ellipses in the nMDS analysis (Fig. 3C), but this conclusion was not supported by the betadisper analysis due to a low sample size (Fig. 4B). However, the PERMANOVA did determine significant differences between Upper and Lower Keys low*-Aquarickettsia* genotypes (*df* = 1, *R*^2^ = 0.39, *F* = 5.18, *P* = 0.012, Table S3).

**Figure 5:**
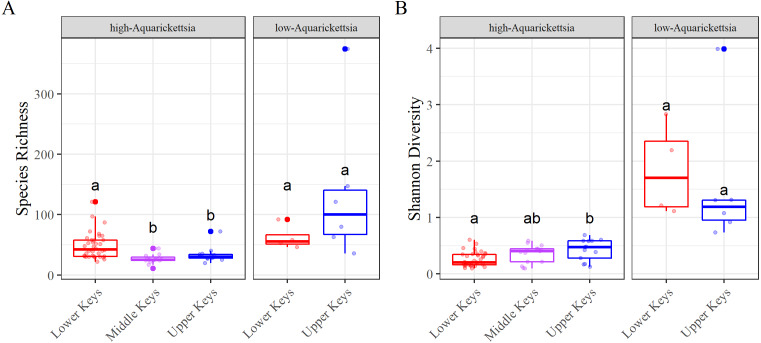
Alpha diversity results measured as species richness and Shannon diversity. Alpha diversity results measured as species richness and Shannon diversity Alpha diversity of the genotypes by high- or low-*Aquarickettsia* and by collection region; (A) Species richness and (B) Shannon diversity. Different letters denote significant differences according to the post hoc pairwise comparison tests.

Alpha diversity was significantly different between high- and low-*Aquarickettsia* genotypes (Richness *P* = 16.7e−5, *df* = 1, *X*^2^ = 14.173; Shannon *P* = 4.2e−7, *df* = 1, *X*^2^ = 25.6; Figs. 5A and 5B). Visualization of the relative abundances of bacteria genera also supported the greater diversity in bacteria of the low-*Aquarickettsia* genotypes (Fig. 2). Species richness (*P* = 2.7e−5, *df* = 2, *X*^2^ = 21.03; Fig. 5A) and Shannon diversity index (*P* = 0.008, *df* = 2, *X*^2^ = 9.55; Fig. 5B) were both significantly different by collection region for the high-*Aquarickettsia* genotypes. Pairwise tests determined that only alpha diversity metrics of the high-*Aquarickettsia* genotypes from the Lower and Upper Keys were significantly different (Richness *P* = 0.017 and Shannon *P* = 0.02). Alpha diversity did not significantly differ between the Upper and Lower Keys low-*Aquarickettsia* genotypes (Richness *P* = 0.2, *df* = 1, *X*^2^ = 1.64; Fig. 5A; Shannon *P* = 0.4, *df* = 1, *X*^2^ = 0.73; Fig. 5B).

### *Aquarickettsia* and *Spirochaeta* are differentially abundant taxa in *A. cervicornis* genotypes from different regions of the Florida Keys

**Figure 6:**
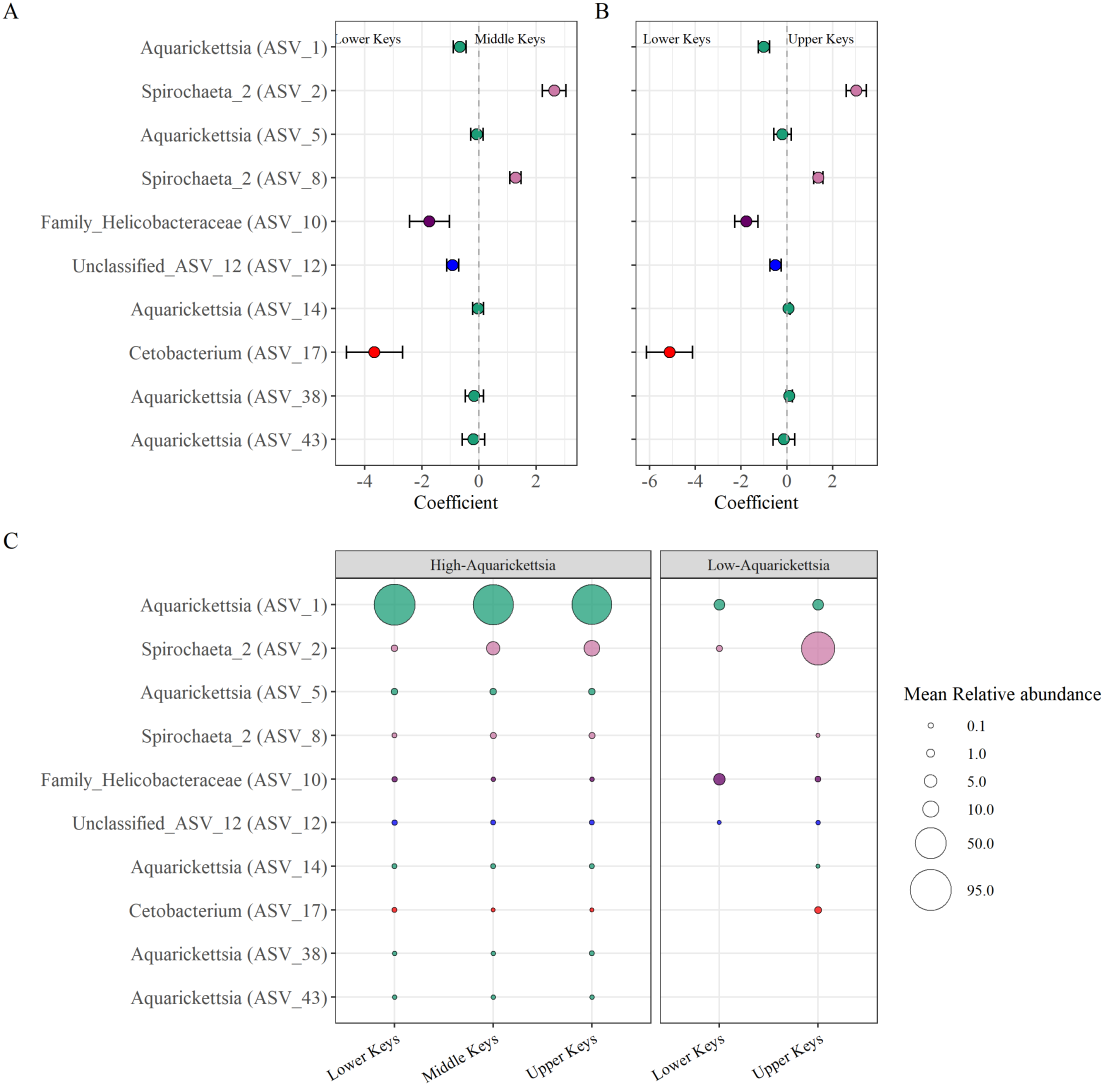
Differentially abundant taxa as a function of collection region. Differentially abundant taxa as a function of collection region. (A) Taxa that were significantly (*P* < 0.05) enriched in the high-*Aquarickettsia* genotypes of the Lower Keys (<0) or in the Middle Keys (>0), and (B) those same taxa significantly enriched in the Lower Keys (<0) or the Upper Keys (>0) high-*Aquarickettsia* genotypes as determined by the beta-binomial regression models used within the corncob package. Coefficients indicate the mean change in relative abundance (±standard error of the mean) of the ASVs. (C) Relative abundances (corresponding with size of the bubble) of the differentially abundant taxa in both the high- and low-*Aquarickettsia* genotypes. Colors correspond to genus as labeled in Fig. 3 unless the genus was not visualized, *i.e.,* Unclassified_ASV_12 (blue) and Cetobacterium (red).

Beta-binomial regression models from the corncob analysis determined ten significantly differentially abundant ASVs as a function of the original collection region for the high-*Aquarickettsia* genotypes (Fig. 6, Table S4). Only two ASVs (two and eight) in the *Spirochaeta* genus were significantly, relatively positively enriched in both the Middle and Upper Keys genotypes (Figs. 6A and 6B). Eight ASVs from four genera were significantly, relatively positively enriched in the Lower Keys, including five *Aquarickettsia* ASVs (ASV 1, 5, 14, 38, and 43). ASV 1, an *Aquarickettsia sp.,* was highly relatively abundant in genotypes from all initial collection regions; however, it was more relatively abundant and less variable in genotypes from the Lower Keys (Fig. S3). Additional taxa significantly, relatively enriched in the high-*Aquarickettsia* genotypes from the Lower Keys include an ASV from the Helicobacteraceae family (ASV 10), an unclassified Proteobacteria ASV (ASV 12), and a *Cetobacterium* sp. (ASV 17). According to NCBI BLAST, Unclassified ASV 12 was most similar to an uncultured bacterium clone p1BB03 found in *A. palmata* from Puerto Rico (E =1e−117, 98.39% identity, GenBank EU861195.1), and also closely matched to an uncultured bacterium clone Acer_J17 in *A. cervicornis* from Bocas del Toro, Panama (E =5e116, 97.98% identity, GenBank GU117990.1; Sunagawa, Woodley & Medina, 2010). Six ASVs were significantly differentially abundant between Lower and Upper Keys low-*Aquarickettsia* genotypes (Table S5 and Fig. S4). A *Spirochaeta* sp. (ASV 2), an *Enterococcus* sp. (ASV 9), unclassified ASV 21, a *Cloacibacterium* sp. (ASV 33), and ASV 35 in the family Microbacteriaceae were relatively positively enriched in the Upper Keys low-*Aquarickettsia* genotypes. Unclassified ASV 21 was most similar to an uncultured marine bacterium clone S82_61c03 found in the skeleton of *Cladocora caespitosa* in the Mediterranean Sea (E =3e−104, 94.49% identity, GenBank JQ235900.1; Meron et al., 2012). Only ASV 10 in the Helicobacteraceae family was relatively positively enriched in the Lower Keys low-*Aquarickettsia* genotypes.

## Discussion

We found significant differences in the microbial communities of *A. cervicornis* based on the initial collection region of the genotypes; however, the putative intracellular bacterial parasite, *Ca.* Aquarickettsia rohweri, dominated most genotypes sampled from all regions. It represented an average of 94.3 ± 5.51% of the microbiome in non-outlier, high-*Aquarickettsia,* genotypes (64 of 74 genotypes sampled). We found that a single ASV from this species was dominant in our samples; there was a 100% match to the published 16S rRNA sequence for the type species, strain *acerv44*, for which the complete genome is available (Klinges et al., 2019). This same strain appears to be dominant across samples of *Acropora cervicornis* throughout the Florida Keys and broader Caribbean region. The 16S rRNA sequence of this strain is identical to the dominant ASV in Florida *A. cervicornis* from Rosales et al. (2019) and to the second most dominant ASV in *A. cervicornis* from the Cayman Islands, which differed by a single nucleotide from the most dominant ASV in these corals (Miller et al., 2020). This sequence was furthermore found to be 99.18% identical to the dominant Rickettsiales ASV in samples of *A. cervicornis* from Puerto Rico in Godoy-Vitorino et al. (2017). Members of *Aquarickettsia* recently identified in mucus of *A. cervicornis* from Mote’s *in situ* nursery and in outplants (Aguirre et al., 2022) ranged in identity to strain *acerv44* from 94–100%. High abundance of this taxon has been proposed as a biomarker for disease susceptibility in *A. cervicornis*, as *Aquarickettsia* has been observed to dominate disease-susceptible genotypes while remaining relatively low in abundance within disease-resistant genotypes (Rosales et al., 2019; Klinges et al., 2020).

It is as of yet unknown how this taxon becomes dominant in microbiomes of *A. cervicornis*, as *Aquarickettsia* is notably absent in early life stages of this species, suggesting that the putative parasite is not inherited vertically (Baker et al., 2022). Genomes of this bacterial genus were found to cluster phylogenetically by collection region, rather than coral host, further supporting environmental acquisition (Baker et al., 2022). Low microbial diversity and single-taxon dominance such as observed in genotypes dominated by *Aquarickettsia* has been linked to disease in human systems, while conversely high diversity is proposed to support greater defenses against pathogens and contribute to host plasticity in the face of environmental change (West et al., 2019; Bourne, Morrow & Webster, 2016). The observed dominance of this taxon across many genotypes of *Acropora cervicornis* and the limited responsivity of microbiomes of this species to transplantation may be reflective of reduced fitness of Caribbean *Acropora* compared to non-Caribbean *Acropora*. Ziegler et al. (2019) found that *A. hemprichii* possessed a flexible, environmentally-responsive microbiome that may allow for greater adaptation to environmental change. Further study is necessary to characterize the effects of low microbial diversity on the health of Caribbean *Acropora* and to ascertain whether low microbial diversity signatures persist when these genotypes are restored to the reef.

ASVs in the genus *Spirochaeta*, two in the high-*Aquarickettsia* and one in the low-*Aquarickettsia* subsets, were significantly differentially abundant in genotypes from the Middle and Upper Keys when compared to the Lower Keys. ASVs in this genus were previously found in high abundances in *A. palmata* (Rosales et al., 2019). No *Spirochaeta* ASVs were found in relative abundances greater than 1% in 16 *A. cervicornis* genotypes from Mote’s *in situ* nursery sampled in 2015, 15 of which were again sampled in 2019 and studied here (all of which were originally collected from the Lower Keys; Klinges et al., 2020). Although *Spirochaeta* sp. are associated with nutrient cycling in a wide range of corals (Lawler et al., 2016; Van de Water et al., 2016; Park et al., 2021), *Spirochaeta* sp. in the microbiome of a disease susceptible *A. cervicornis* genotype did not respond to nutrient enrichment*,* while relative abundances of *Aquarickettsia sp.* significantly increased (Klinges et al., 2022). Lower abundance taxa that were still significantly relatively, positively enriched in the genotypes originally collected in the Lower Keys included ASVs in the Helicobacteraceae family, a *Cetobacterium* sp., and an unclassified Proteobacteria ASV similar to ASVs found previously in other *Acropora sp.* samples. Members of the Helicobacteraceae family were enriched in *A. cervicornis* with white band disease in Panama (Gignoux-Wolfsohn, Aronson & Vollmer, 2017; Gignoux-Wolfsohn et al., 2020).

Several genotypes in the Upper and Lower Keys had distinct microbiomes, as apparent from the nMDS and outlier detection analysis, and were termed “low-*Aquarickettsia*” because of their high bacterial diversity and low abundances of *Aquarickettsia* ASVs. These low-*Aquarickettsia* genotypes had significantly higher richness and Shannon diversity than the high-*Aquarickettsia* genotypes. Like those of the high-*Aquarickettsia* genotypes, the microbial communities of the low-*Aquarickettsia* genotypes significantly differed by initial collection region. According to the differential abundance analysis, these differences were due to six ASVs. These included two ASVs that were significantly, relatively differentially abundant in the high-*Aquarickettsia* genotypes: a *Spirochaeta* ASV was positively enriched in the Upper Keys while a Helicobacteraceae ASV was positively enriched in the Lower Keys’ low-*Aquarickettsia* genotypes. The high bacterial diversity of these low-*Aquarickettsia A. cervicornis* genotypes is indicative of potential disease resistance, as it has been proposed that the comparatively high diversity in these genotypes may occlude niche space that could otherwise be infiltrated by opportunistic species (Klinges et al., 2020). Our analysis included 15 out of 16 genotypes used in this previous study of coral fragments collected in 2015 (Klinges et al., 2020). However, microbiome analysis of collections from 2019 (the present study) show that genotypes M-L-3 and M-L-7 (genotypes 3 and 7 in Klinges et al., 2020), while found to be comparatively low in *Aquarickettsia* at both timepoints, now are dominated by *Spirochaeta* and an unclassified ASV, respectively, rather than dominated by no single taxon as found in 2015 samples of these genotypes. This reduction in community evenness from 2015 to 2019 may reflect either a reduction in capacity for disease resistance resulting from a decrease in microbial diversity over time, or instead the development of a new symbiotic relationship with *Spirochaeta* and an unclassified species that may confer unknown benefits to these genotypes of *A. cervicornis* lacking *Ca.* Aquarickettsia rohweri.

Coral host-microbe interactions are influenced by environmental factors that vary over both space and time (Dinsdale et al., 2008; Dunphy et al., 2019), even over small spatial scales (Wainwright et al., 2019). Microbiomes of both high and low-*Aquarickettsia* genotypes significantly differed by initial collection region. Pairwise comparisons of diversity, both alpha and beta, of the high-*Aquarickettsia* genotypes indicated that the differences by initial collection region were driven by significant differences between genotypes from the Upper and Lower Keys. Interestingly, the microbial communities of the genotypes initially collected from the Middle Keys were more similar to the Upper Keys than to the Lower Keys. This pattern of dissimilarity in *A. cervicornis* microbiomes across the Florida Keys supports the existence of geographic influences on host-microbe interactions, which may be retained for years after relocation. The distinct spatial regions of FCR experience pronounced environmental differences (Jaap, 1984; Klein III & Orlando Jr, 1994; Murdoch & Aronson, 1999) that likely play a role in structuring the differences in the microbiomes seen here. However, *A. cervicornis* from the different regions are also genetically distinct (Drury, Manzello & Lirman, 2017), thus it is difficult to tease apart the role of host genetics from that of the environment when comparing the microbiomes of *A. cervicornis* across geographical gradients in the Florida Keys. Regardless, genotype, environmental factors (Drury, Manzello & Lirman, 2017), and microbes (Mao-Jones et al., 2010; Ritchie, 2011) have considerable influence on the health and survival of their hosts, therefore geographical differences in initial collection region should be considered by managers and practitioners when determining outplanting strategies, even on a regional (*i.e.,* Florida Keys) scale.

The rate at which the coral microbiome responds to changing environments may play a role in coral resilience. Rapid shifts in microbiome structure in response to environmental shifts may reflect a highly adaptive microbiome that may lead to host environmental flexibility, but is at a higher risk for the loss of beneficial species as well as opportunists (Ziegler et al., 2019). In contrast, corals that possess an inflexible microbiome may be slow to respond to environmental cues, but may preserve relationships with essential members of the microbiome that perform key functions (Ziegler et al., 2019). Interestingly, although the genotypes originally collected from the Middle and Upper Keys had been located in Mote’s *in situ* nursery in the Lower Keys for an average of 3.4 years, their microbiomes still held geographic markers, suggesting the potential for retaining microbiome signatures over time. Host identity is known to have greater control on microbiome composition than temporal differences (Dunphy et al., 2019), however, there is still evidence of temporal variability in coral microbiomes (Epstein et al., 2019). Stability of coral microbiomes over time or when transplanted is known to be a species-specific response (Roitman et al., 2020; Deignan & McDougald, 2021; Dunphy, Vollmer & Gouhier, 2021; Strudwick et al., 2022). In a previous study of microbiome shifts resulting from translocation, a non-Caribbean Acroporid coral, *A. hemprichii,* experienced significant microbiome restructuring when moved across sites in the Red Sea (Ziegler et al., 2019). In contrast, Dunphy, Vollmer & Gouhier (2021) found that *A. cervicornis* in Panama had highly stable microbiomes during a reciprocal field transplant experiment that also perturbed the microbiome using an antibiotic. As we did not sample the genotypes studied here before they were moved to Mote’s nursery, we do not know the structure of their microbiomes at the time of collection or soon after translocation. Further research is therefore needed to understand how microbiomes of restored corals may change through time—when moved from the reef to a nursery, transferred among different nurseries, or outplanted from a nursery back onto the reef. The presence of geographic signatures of microbiome composition in our study suggest that Floridian genotypes of *Acropora cervicornis* may possess a microbiome signature associated with geographic region of origin, which may result in preserved relationships with potential symbionts.

## Conclusions

The present study characterized the microbiomes of apparently healthy *Acropora cervicornis* genotypes that originated from different regions across Florida’s Coral Reef and have been housed in Mote Marine Laboratory’s *in situ* nursery near Looe Key, FL (USA) for multiple years. We found significant differences in bacterial diversity of the genotypes driven by the initial collection region, even after the corals had been kept under common garden conditions for multiple years. These differences in microbial communities due to geographical origin most likely reflect the genetic relatedness and environmental history of the genotypes sampled. Our results suggest that the abundance of two key bacterial taxa, *Ca.* Aquarickettsia and *Spirochaeta*, may play an important role in distinguishing bacterial communities among *A. cervicornis* populations. Most genotypes were dominated by *Aquarickettsia sp.*, a presumptive bacterial parasite of *A. cervicornis* that is associated with disease susceptibility (Klinges et al., 2019; Klinges et al., 2020). Furthermore, several genotypes originally collected from both the Lower and Upper Keys had higher bacterial diversity due to lower abundances of *Aquarickettsia* sp. These low-*Aquarickettsia* genotypes may be a potential resource for restoration initiatives since populations that are more likely to succeed and survive in stressful environments should be thoughtfully targeted for restoration efforts.

## Supplemental Information

10.7717/peerj.13574/supp-1 Supplemental Information 1 Supplemental Tables

## Additional Information and Declarations

### DNA Deposition

The following information was supplied regarding the deposition of DNA sequences: The 16S rRNA sequences are available at NCBI: PRJNA769275.

### Data Availability

The following information was supplied regarding data availability: The data and code are available at GitHub: https://github.com/MoteCHaDlab/FLKeys_AcerMicrobiome_geographicaldifferences. The 16S rRNA sequences are available at NCBI: PRJNA769275.
