# Supplementary material for: Geographically driven differences in microbiomes of Acropora cervicornis originating from different regions of Florida’s Coral Reef
Source: PeerJ. 2022 Jun 16;10:e13574. doi: 10.7717/peerj.13574 (PMC9206844; doi:10.7717/peerj.13574)

**Geographically driven differences in microbiomes of *Acropora cervicornis* originating from different regions of Florida’s Coral Reef**

Sara D. Williams^1^, J. Grace Klinges^2^, Samara Zinman^1^, Abigail S. Clark^2,3^, Erich Bartels^2^, Marina Villoch Diaz-Mauriño^2^, Erinn M. Muller^1^

^1^ Mote Marine Laboratory, Sarasota, Florida, USA

^2^ Mote Marine Laboratory, Elizabeth Moore International Center for Coral Reef Research and Restoration, Summerland Key, Florida, USA

^3^ The College of the Florida Keys, Key West, Florida, USA

**Supplemental Material**

Upon publication, all data and code are available at <https://github.com/MoteCHaDlab/FLKeys_AcerMicrobiome_geographicaldifferences>.

**Table S1:** Information on number of genotypes from each region and time the genotypes had spent in the same nursery before sampling in 2019. See the section below for further temporal analyes.

| Region | Lower keys | Middle keys | Upper keys |
| --- | --- | --- | --- |
| Number of genotypes | 40 | 15 | 19 |
| Mean ± SE time in nursery (Years) | 8.1±0.5 | 3.4±0.2 | 3.3±0.2 |
| Median ±  SE time in nursery (Years) | 9.4±0.5 | 3.5±0.2 | 3.5±0.2 |

**Table S2.** Table of reads retained during the bioinformatic pipeline.

| Initial number of reads | 24,392,378 |
| --- | --- |
| Number of reads after filter and trim step in DADA2 | 21,770,005 |
| Number of reads after removing any ASVs that were considerably off target length | 19,989,462 |
| Number of reads after chimeras were removed | 19,821,934 |
| Number of reads after chloroplasts (17,363) removed | 19,804,571 |
| Number of reads after mitochondria (82) removed | 19,804,489 |
| Number of reads after eukaryotes (56) removed | 19,804,433 |
| Final number of reads after the pruning step removed taxa with less than 10 reads in 10% of the samples (equated to 4,641 reads) | **19,799,792** |

**Table S3.** Full PERMANOVA results for all datasets and results of the multiple PERMANOVA tests with Bonferroni adjusted p-values for the full and high*-Aquarickettsia* datasets.

| Dataset | Degrees of freedom / Residuals | F-statistic | R^2^ | p-value | Multiple Comparisons | Adjusted p-value |
| --- | --- | --- | --- | --- | --- | --- |
| Full dataset | 2 / 71 | 6.464 | 0.154 | 0.002 | Middle & Upper | 0.030 |
|  |  |  |  |  | Lower & Upper | 0.012 |
|  |  |  |  |  | Lower & Middle | 0.177 |
| Low-*Aquarickettsia* | 1 / 8 | 5.176 | 0.393 | 0.011 | NA | NA |
| High-*Aquarickettsia* | 2 / 61 | 0.046 | 0.339 | 0.001 | Middle & Upper | 0.555 |
|  |  |  |  |  | Lower & Upper | 0.003 |
|  |  |  |  |  | Lower & Middle | 0.003 |

**Table S4.** Results of the corncob differential abundance analysis for the non-outlier dataset, the high-*Aquarickettsia* genotypes: coefficients associated with abundance. Note that for ASV 5, the intercept was significant (P <0.001) and the coefficients associated with dispersion (not listed, see code for values) were significant.

| ASV | Genus | Lower vs. Middle Keys | | | Lower vs. Upper Keys | | |
| --- | --- | --- | --- | --- | --- | --- | --- |
|  |  | Estimate ± S.E. | t value | P value | Estimate ± S.E. | t value | P value |
| 1 | *Aquarickettsia* | -0.67±0.2 | -3.02 | 0.004 | -1.0±0.2 | -4.15 | < 0.001 |
| 2 | *Spirochaeta_2* | 2.64±0.4 | 6.33 | <0.001 | 3.05±0.4 | 6.85 | <0.001 |
| 5 | *Aquarickettsia* | -0.07±0.2 | -0.33 | 0.739 | -0.19±0.4 | -0.49 | 0.623 |
| 8 | *Spirochaeta_2* | 1.28±0.2 | 6.53 | <0.001 | 1.38±0.2 | 6.72 | <0.001 |
| 10 | Family Helicobacteraceae | -1.73±0.7 | -2.48 | 0.016 | -1.77±0.5 | -3.53 | <0.001 |
| 12 | Unclassified | -0.92±0.2 | -4.40 | <0.001 | -0.50±0.2 | -1.99 | 0.0512 |
| 14 | *Aquarickettsia* | -0.03±0.2 | -0.15 | 0.882 | -0.07±0.1 | 1.05 | 0.299 |
| 17 | *Cetobacterium* | -3.66±1.0 | -3.72 | <0.001 | -5.12±1.0 | -5.11 | <0.001 |
| 38 | *Aquarickettsia* | -0.16±0.3 | -0.51 | 0.614 | 0.11±0.1 | 0.85 | 0.401 |
| 43 | *Aquarickettsia* | -0.20±0.4 | -0.50 | 0.616 | -0.12±0.5 | -0.26 | 0.797 |

**Table S5.** Results of the corncob differential abundance analysis for the outlier dataset, the low-*Aquarickettsia* genotypes: coefficients associated with abundance.

| ASV | Genus | Lower vs. Upper Keys | | |
| --- | --- | --- | --- | --- |
|  |  | Estimate ± S.E. | t value | P value |
| 2 | *Spirochaeta_2* | 5.72±0.6 | 9.24 | <0.001 |
| 9 | *Enterococcus* | 7.24±1.7 | 4.25 | 0.005 |
| 10 | Family Helicobacteraceae | -2.93±0.9 | -3.41 | 0.014 |
| 21 | Unclassified | 7.07±2.1 | 3.26 | 0.017 |
| 33 | *Cloacibacterium* | 6.82±1.6 | 4.40 | 0.005 |
| 35 | Family Microbacteriaceae | 6.35±1.7 | 3.83 | 0.009 |


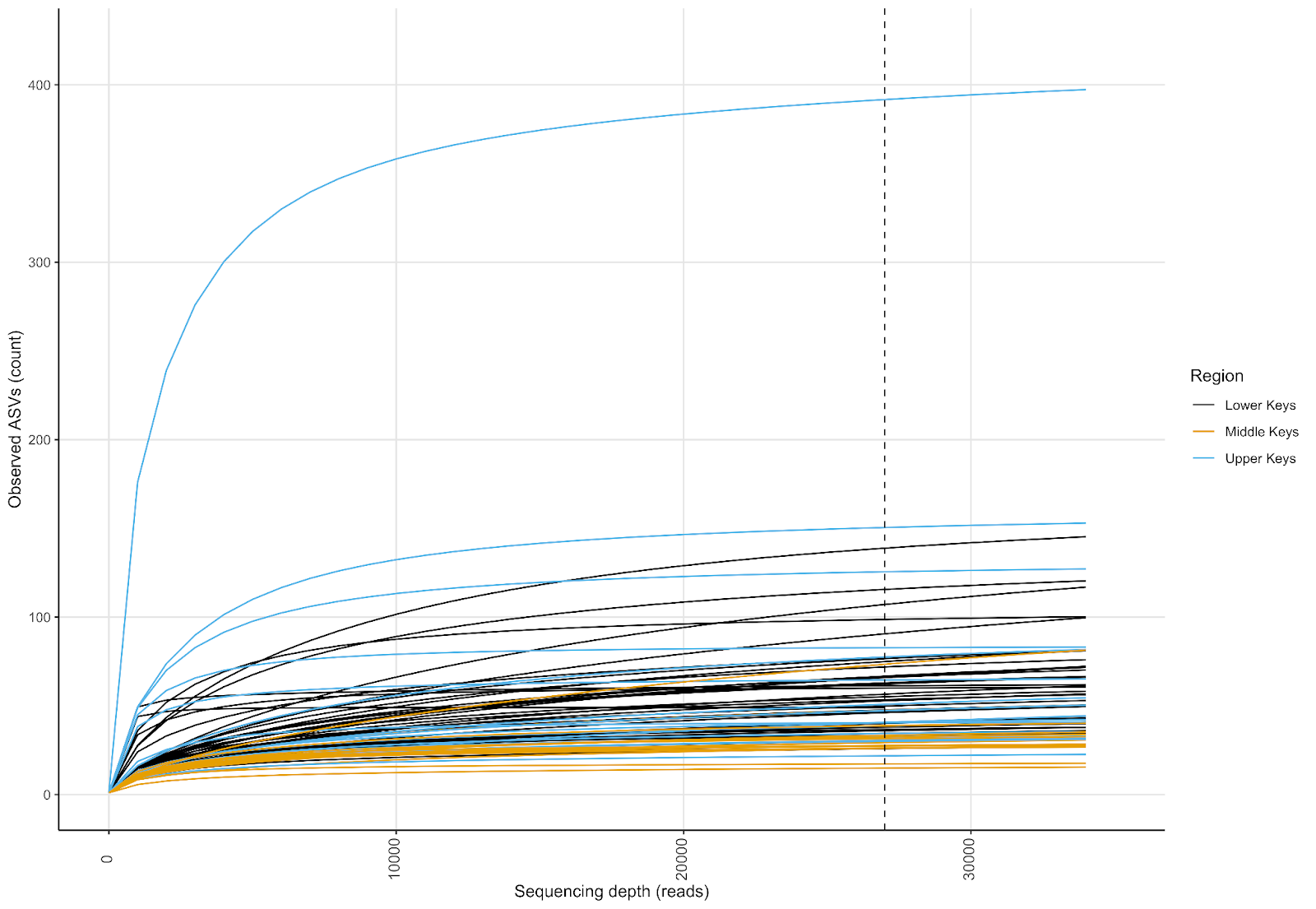


**Figure S1**: Rarefaction curves produced by command amp_rarecurve() in program ampvis2 showing sample diversity (as observed ASVs) captured at a rarefaction depth of 26,537 (minimum read depth across dataset).

**
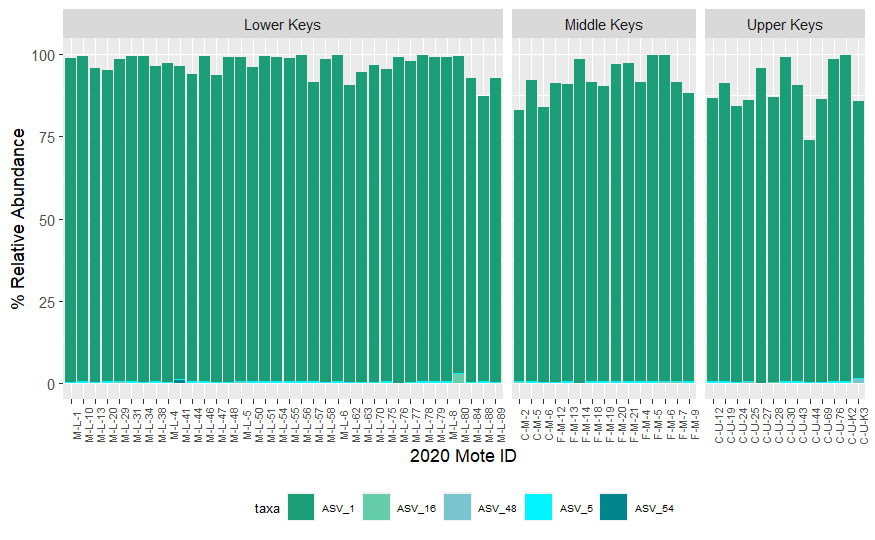

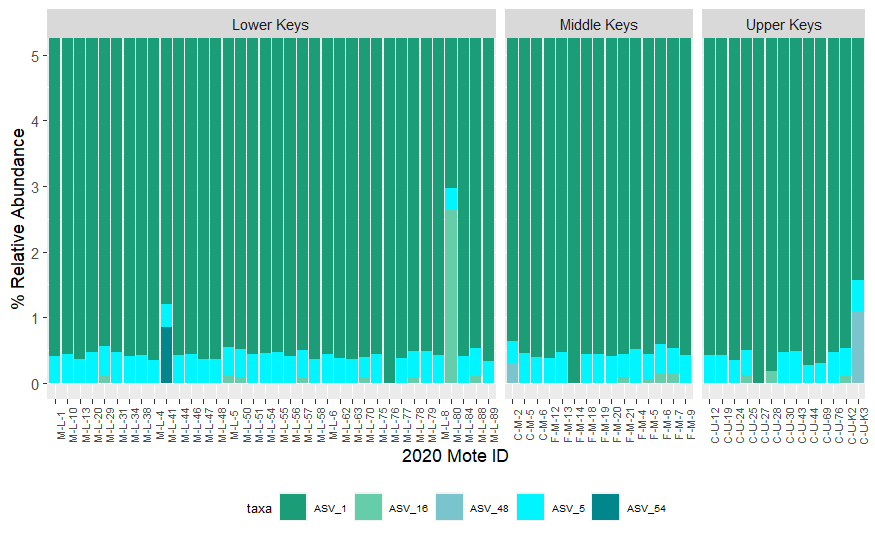
**

**Figure S2.** Relative abundances of the dominant *Aquarickettsia sp*. ASVs in the high-*Aquarickettsia* genotypes. Top panel shows the full relative abundance range (0-100%) and the bottom panel shows just the bottom 5%.


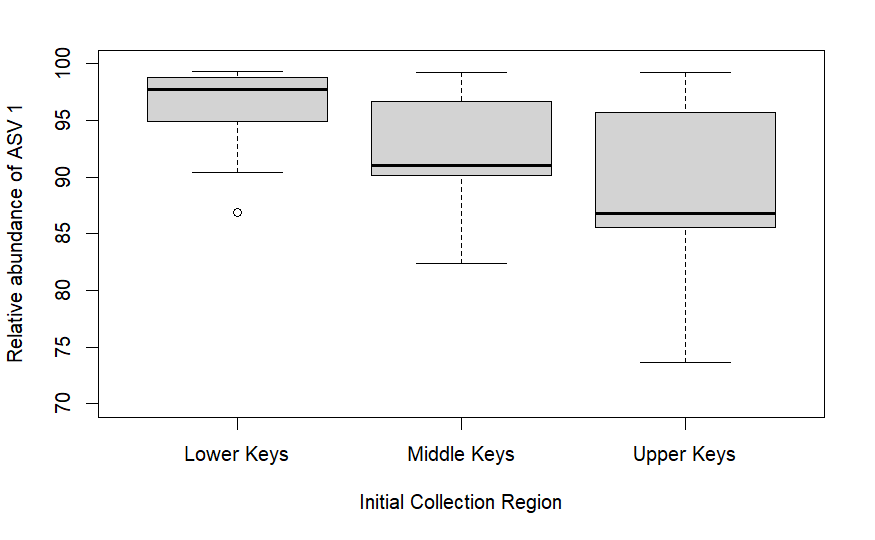


**Figure S3**. Relative abundances of ASV 1 (*Aquarickettsia sp.*) in genotypes from the different initial collection regions.

**
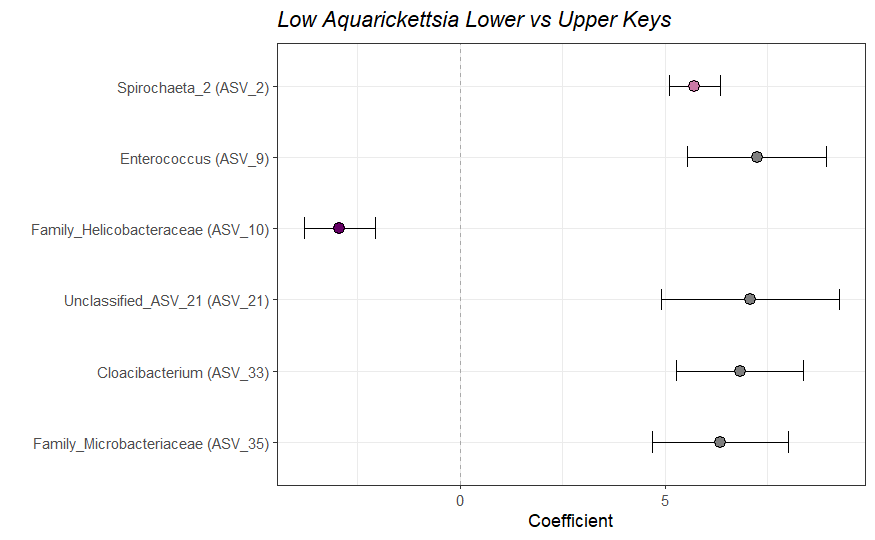
**

**Figure S4.** Differentially abundant taxa as a function of the collection region for the low-*Aquarickettsia* genotypes. Taxa that were significantly (P<0.05) relatively enriched in the high-*Aquarickettsia* genotypes of the Lower Keys (coefficient < 0) or in the Upper Keys (Coefficient > 0). Coefficients indicate the mean change in relative abundance (± standard error of the mean) of the ASVs.

***Supplemental Methods & Results: Diversity as a Function of Time Spent in the Nursery***

This study was not designed to look at temporal patterns of the microbiomes as the genotypes were all moved to the nursery at different times and we do not have samples from before the frags were moved. However, we do have the data on how long each genotype had been in the nursery at the time of microbiome sampling for this study. Mean and medium times spent in the nursery are listed in Table S1. Genotypes from the Lower Keys had been in the nursery (which is located in the Lower Keys) 0.2 to 11.8 years. Genotypes from the Upper Keys had been in the nursery 1.4 to 4 years. Genotypes from the Middle Keys had been in the nursery 1.5 to 4 years.

The time spent in the nursery significantly differed by initial collection region of the genotypes (Kruskal Wallis rank sum test, chi-squared=35.99, df=2, P=1.53e-08). Genotypes originally from the Upper and Middle Keys had not been in the nursery a significantly different amount of time (Pairwise Kruskal-Wallis, bonferroni adjusted P=0.8); however, those from the Lower Keys had spent significantly different time periods in the nursery than the other two regions(Pairwise Kruskal-Wallis, bonferroni adjusted P<0.001 for both comparisons). Most initial genotypes used for propagation in the nursery were sourced from the Lower keys, and the Genotypes from the Upper and Middle Keys were added later during a coordinated ‘swap’ with other nurseries in those regions. The time spent in the nursery by outlier genotypes (low-*Aquarickettsia*) was not significantly different than that of the non-outlier (high-*Aquarickettsia*) genotypes (Kruskal-Wallis rank sum test, chi-squared=2.5, df=1, P=0.11).

We used a series of linear regressions to determine if diversity varied temporally for either the Lower Keys or the grouped Middle and Upper Keys genotypes. Only richness significantly increased over time in genotypes originally collected from the Lower Keys (Table S6). Genotypes from the Lower Keys have the widest range of time spent in the nursery. While it is interesting that richness has a positive relationship with time spent in the nursery by Lower Keys genotypes, we cannot definitively say that it increases with time spent in the nursery because we do not have initial samples from these genotypes and thus do not have a baseline. Future studies should consider potential long-term variation in coral microbiome diversity in relation to coral restoration and the coral “stock” held in nurseries used for propagation and outplanting.

**Table S6**. Results of linear regressions for temporal analyses

| Diversity Metric | Region | Transformation used if needed | DF | Adjusted R-squared | P-value |
| --- | --- | --- | --- | --- | --- |
| Betadisper model distances | Middle & Upper | NA | 22 | 0.02 | 0.25 |
|  | Lower | 1/sqrt | 34 | 0.003 | 0.30 |
| Shannon diversity index | Middle & Upper | NA | 22 | -0.02 | 0.50 |
|  | Lower | Log | 34 | -0.03 | 0.97 |
| Richness | Middle & Upper | 1/Log | 22 | 0.02 | 0.24 |
|  | Lower | Log | 34 | 0.28 | 0.0005 |

***Supplemental Method & Results: Heat Trees of Differential Abundance***

     Heat trees of bacterial abundances and phylogenetic relatedness were built from the microbiome relative abundance data using the metacoder package R (metacoder package, Foster et. al, 2017). The heat tree matrix (Fig. S5A) displays differences between regions based off of the log2 median ratios of abundances in one region over the other (Fig. S5B shows the taxonomy of the nodes). These results are similar to the corncob differential abundance analysis results -- they show what taxa are driving the difference between regions;  however, they also show the differences on a finer taxonomic level. Again, *Spirochaeta sp.* were much more abundant in the Upper and Middle Keys than in the Lower Keys genotypes. Different taxa are seen here (Fig. S5) because the full dataset, high and low -*Aquarickettsia* genotypes, was used, so the heat tree matrix also captures the differentially abundant taxa from the outliers. In the Lower versus Middle Keys heat tree (top left of Fig. S5A), the taxa driving the differences between regions are more abundant in the Lower Keys, except *Spirochaeta*. In the Upper versus Lower Keys heat tree (top right of Fig. S5A), more of the taxa driving the differences are less differentially abundant; but of the ones that are, *Spirochaeta sp.* are more abundant in the Upper Keys and *Aquarickettsia sp*. are more abundant in the Lower Keys. All of the taxa driving the differences between the Upper and Middle Keys (bottom right of Fig. S5A) are more abundant in the Upper Keys.

Foster ZSL, Sharpton TJ, Grünwald NJ (2017) Metacoder: An R package for visualization and manipulation of community taxonomic diversity data. PLOS Computational Biology 13(2): e1005404.<https://doi.org/10.1371/journal.pcbi.1005404>

**Figure S5 A & B.** The first plot (A) is a heat tree matrix, with size and intensity of color scaling to represent abundance (i.e. the bigger the node, the more abundant it is). The matrix displays how the diversity and abundance of the *A. cervicornis* microbiomes differ by region. The darker brown the nodes are displayed, the more abundant it is in the region represented by the column. Likewise, the darker blue-green the nodes are displayed, the more abundant the node is in the region represented by the row. The second plot contains the labels explaining what each of the nodes in the heat tree matrix represent.**
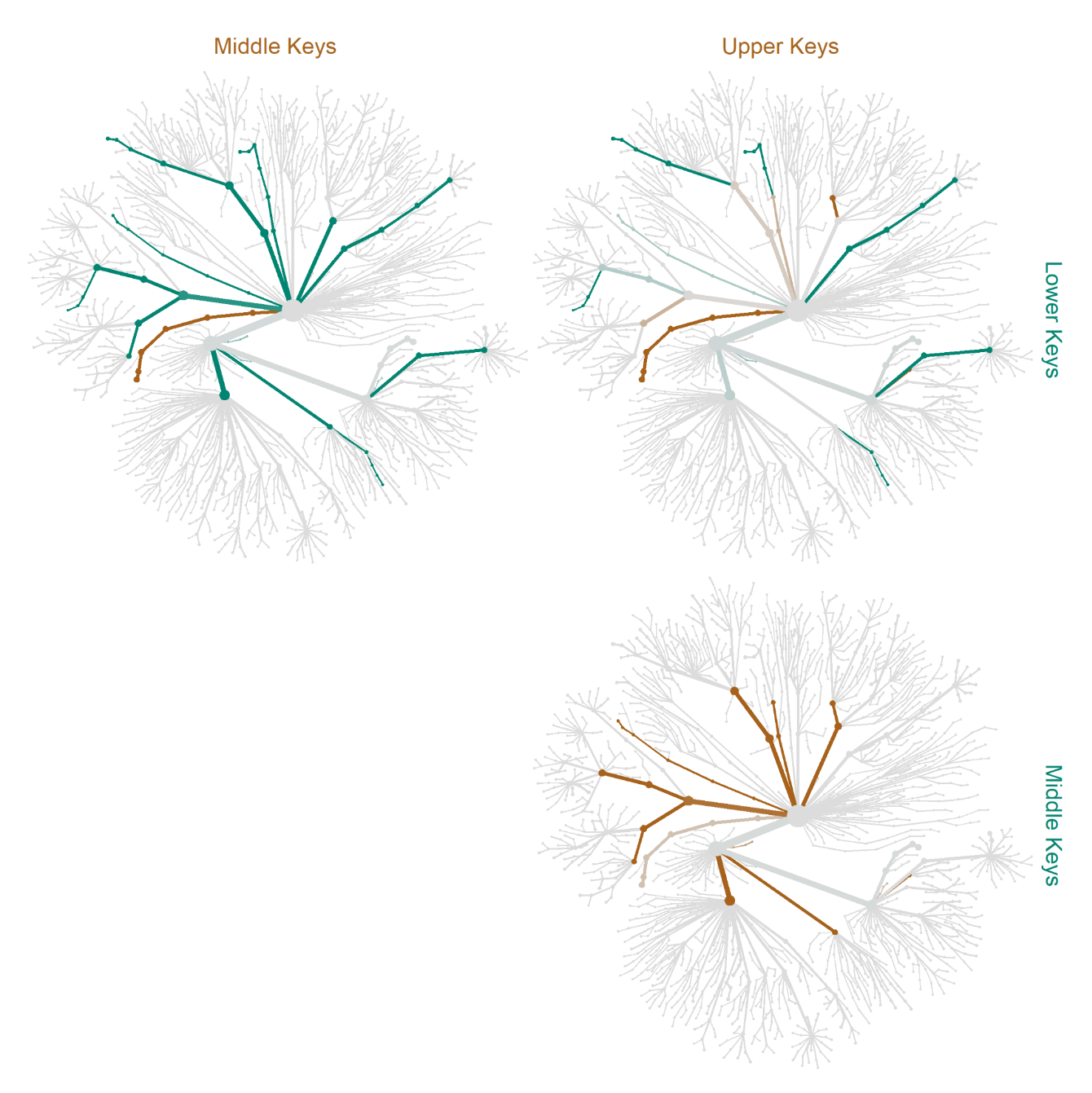
**
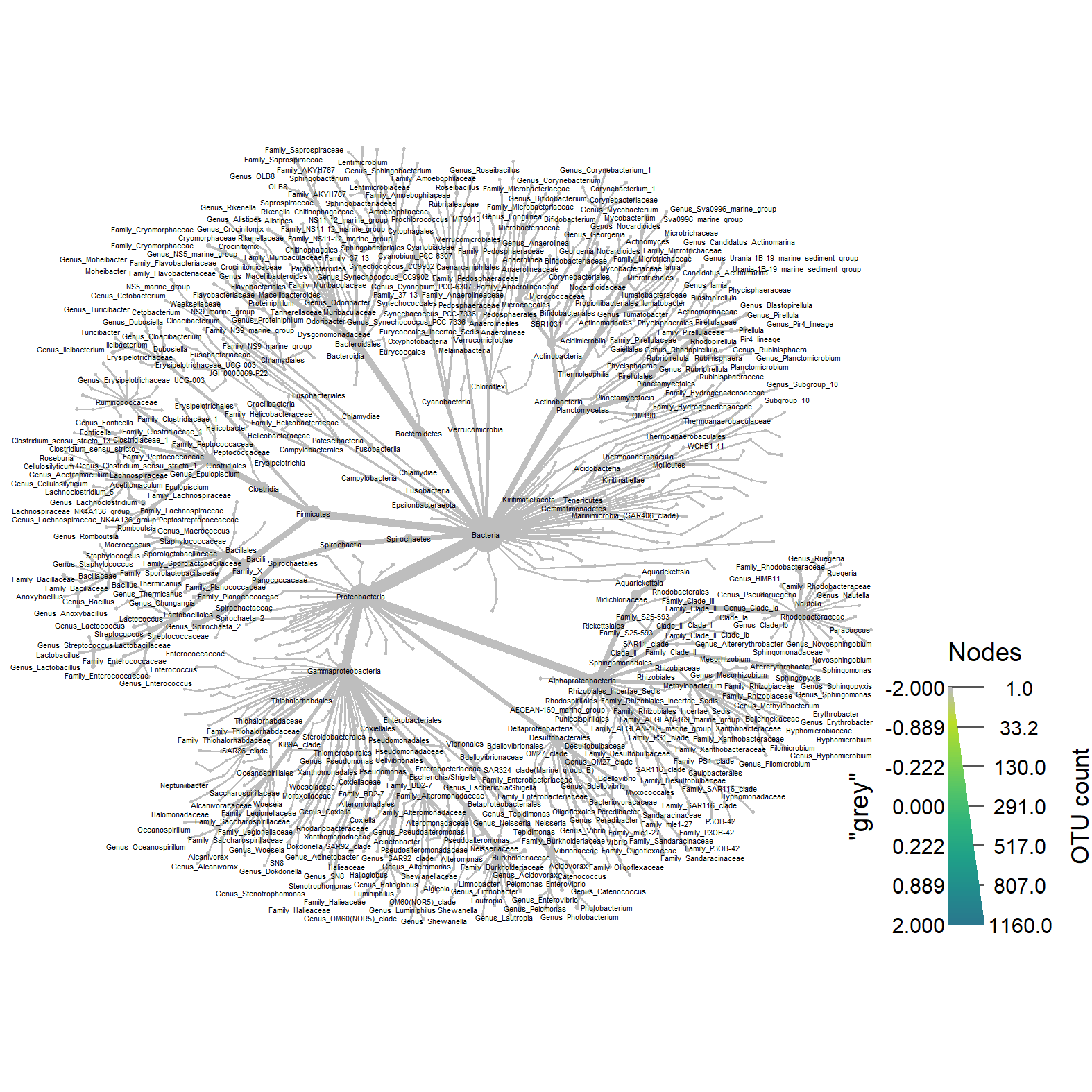

Supplement: Supplemental Information 1 [file peerj-10-13574-s001.docx]
